# Anthocyanin biosynthetic genes in *Brassica rapa*

**DOI:** 10.1186/1471-2164-15-426

**Published:** 2014-06-04

**Authors:** Ning Guo, Feng Cheng, Jian Wu, Bo Liu, Shuning Zheng, Jianli Liang, Xiaowu Wang

**Affiliations:** Institute of Vegetables and Flowers, Chinese Academy of Agricultural Sciences, Zhongguancun Nandajie No.12, Haidian district Beijing, 100081 P. R. China

**Keywords:** Comparative genomics, Anthocyanin biosynthetic genes, Whole genome duplication, *Brassica rapa*, Cruciferae

## Abstract

**Background:**

Anthocyanins are a group of flavonoid compounds. As a group of important secondary metabolites, they perform several key biological functions in plants. Anthocyanins also play beneficial health roles as potentially protective factors against cancer and heart disease. To elucidate the anthocyanin biosynthetic pathway in *Brassica rapa*, we conducted comparative genomic analyses between *Arabidopsis thaliana* and *B. rapa* on a genome-wide level.

**Results:**

In total, we identified 73 genes in *B. rapa* as orthologs of 41 anthocyanin biosynthetic genes in *A. thaliana*. In *B. rapa*, the anthocyanin biosynthetic genes (ABGs) have expanded and most genes exist in more than one copy. The anthocyanin biosynthetic structural genes have expanded through whole genome and tandem duplication in *B. rapa*. More structural genes located upstream of the anthocyanin biosynthetic pathway have been retained than downstream. More negative regulatory genes are retained in the anthocyanin biosynthesis regulatory system of *B. rapa.*

**Conclusions:**

These results will promote an understanding of the genetic mechanism of anthocyanin biosynthesis, as well as help the improvement of the nutritional quality of *B. rapa* through the breeding of high anthocyanin content varieties.

**Electronic supplementary material:**

The online version of this article (doi: 10.1186/1471-2164-15-426) contains supplementary material, which is available to authorized users.

## Background

Flavonoids comprise a major group of secondary metabolites, which exhibit a wide range of biological functions in plants [1, 2]. Anthocyanin pigments and flavonol co-pigments are the two major flavonoid compounds, which serve as attractants of pollinators and seed dispersers. They also play an important role in protecting plants against abiotic and biotic stresses [3]. Anthocyanins, like other flavonoid compounds, are known as potent antioxidants [4]. The beneficial health roles of anthocyanins have received considerable attention as they are potentially protective factors against cancer and heart disease [5]. Therefore, a comprehensive understanding of anthocyanin biosynthesis is important for developing foods that are rich in anthocyanins to meet the increasing demand for health-promoting components in our daily diet.

The biosynthetic pathways of anthocyanins have been well characterized [6] and the corresponding genes have been isolated from various plants. In the model plant *A. thaliana*, the biosynthesis, regulation and transport of anthocyanins, specifically most of the structural genes and regulatory proteins involved in anthocyanin synthesis, have been identified and functionally characterized in the last two decades [7–9]. These researches have played important roles in the comprehensive understanding of anthocyanin biosynthesis and revealed the accumulation and metabolic profiles of anthocyanins in *A. thaliana*.

*Brassica rapa* comprises a variety of vegetables, among which Chinese cabbage (*Brassica rapa* L. ssp. *pekinensis*) and pakchoi (*Brassica rapa* L. ssp. *chinensis*) are the two most consumed vegetables in China and throughout East Asia. *B. rapa* vegetables provide dietary fiber, vitamin C, and anti-cancer glucosinolates [10], and are also a potentially important source of dietary flavonols [11]. Several varieties of *B. rapa* are red or purple, such as some kinds of pakchoi, turnip (*Brassica rapa* ssp. *rapa*) and Zicaitai (*Brassica rapa* L. ssp. *chinensis* var. *purpurea*). The purple pigments in *B. rapa* have been identified as anthocyanins [12]. Nevertheless, the genetic mechanism of this purple phenotype is unclear. Because of the previous absence of genome information, little is known about the genes involved in the anthocyanin biosynthetic pathways in *B. rapa*[13, 14]. A complete understanding of the structural and regulatory genes involved is very important for elaborating the mechanism of anthocyanin biosynthesis in *B. rapa*, as well as for the breeding of new *B. rapa* varieties rich in anthocyanins. With the reference genome and gene annotation information for *B. rapa* ‘Chiifu’ [15], we now have the chance to systematically study the anthocyanin biosynthetic genes (ABGs) in *B. rapa*.

Both *B. rapa* and *A. thaliana* belong to the cruciferae family. *B. rapa* (A genome) has undergone whole genome triplication since its divergence from *A. thaliana*, followed by extensive gene loss [15, 16]. In *B. rapa*, the level of gene loss among the three subgenomes is unequal, with one subgenome, the Least Fractionated (LF) subgenome, having retained roughly 70% of its genes during fractionation after triplication, while the other two subgenomes, termed the Medium and Most Fractionated (MF1 and MF2, respectively) subgenomes, have retained much fewer genes [15, 16].

Whole genome duplication (WGD) is a major way of gene copy number expansion in plants. As reported previously, gene families expanded by WGD can maintain the proper balance in biological networks or cascades [17]. After WGD and subsequent gene fractionation, the number of genes that respond to abiotic and biotic stresses or with membrane protein functions tends to be increased [18]. In *B. rapa*, the genes expanded through whole genome triplication (WGT) tend to come from functional categories such as transcriptional regulation, ribosomes, response to abiotic or biotic stimuli, response to hormonal stimuli, cell organization, and transporter functions [15]. However, no detailed information about the status of ABGs after the WGT in *B. rapa* has been available till now. To obtain comprehensive information on the anthocyanin biosynthetic pathway in *B. rapa* and look into the effect of WGT on these genes, comparative genomic analysis between *B. rapa* and *A. thaliana* was performed here based on the reference genome of *B. rapa*[15]. We present several interesting observations about the evolutionary history of ABGs in *B. rapa*, the similarities between the anthocyanin biosynthetic pathways in *A. thaliana* and *B. rapa*, the synteny of ABGs between *A. thaliana* and *B. rapa*, and the expansion and retention of ABGs in *B. rapa*. The results of our systematic analysis of the complete set of ABGs in *B. rapa* will promote the understanding of the genetic mechanism of anthocyanin biosynthesis and the anthocyanin profiles/accumulation in *B. rapa* crops.

## Results and discussion

### ABGs in *B. rapa* identified by comparative genomic analysis

The ABGs have expanded in the genome of *B. rapa*. In *A. thaliana*, 41 ABGs have been reported, including 24 structural genes that encode anthocyanin biosynthesis enzymes, 16 regulatory genes encoding transcriptional factors, and one transport gene that is required for anthocyanin transportation (Table 1; Additional file 1: Table S1). Based on a combination of syntenic and non-syntenic homology analysis, 73 *B. rapa* anthocyanin biosynthetic genes (BrABGs) were identified, representing homologs of 39 out of the 41 AtABGs. The other two AtABGs were not found in *B. rapa*.

**Table 1:** **Anthocyanin biosynthetic genes (ABGs) identified in** ***B. rapa***

***A. thaliana***	***B. rapa***			
	Synteny orthologs			Non-synteny orthologs
	LF	MF1	MF2	
**Structural genes**				
*Biosynthetic genes in phenylpropanoid pathway*				
***AtPAL1*** (AT2G37040)	***BrPAL1.1*** (Bra005221)	***BrPAL1.2*** (Bra017210)	-	-
***AtPAL2*** (AT3G53260)	***BrPAL2.1*** (Bra006985)	***BrPAL2.2*** (Bra039777)	***BrPAL2.3*** (Bra003126)	-
***AtPAL3*** (AT5G04230)	-	-	***BrPAL3.1*** (Bra028793)	***BrPAL3.2*** (Bra030322)
***AtPAL4*** (AT3G10340)	***BrPAL4*** (Bra029831)	-	-	-
***AtC4H*** (AT2G30490)	***BrC4H1*** (Bra018311)	***BrC4H2*** (Bra021636)^*a*^	***BrC4H4*** (Bra022802)	-
		***BrC4H3*** (Bra021637)	***BrC4H5*** (Bra022803)	
***At4CL1*** (AT1G51680)	-	-	***Br4CL1*** (Bra030429)	-
***At4CL2*** (AT3G21240)	***Br4CL2.1*** (Bra031262)	-	-	-
	***Br4CL2.2*** (Bra031263)			
	***Br4CL2.3*** (Bra031265)			
	***Br4CL2.4*** (Bra031266)			
***At4CL3*** (AT1G65060)	***Br4CL3*** (Bra004109)	-	-	-
***At4CL5*** (AT3G21230)	*-*	-	***Br4CL5.1*** (Bra001819)	-
			***Br4CL5.2*** (Bra001820)	
*Early biosynthetic genes*				
***AtCHS*** (AT5G13930)	***BrCHS1*** (Bra008792)	***BrCHS2*** (Bra006224)	***BrCHS*** *3* (Bra023441)	***BrCHS4*** (Bra036307)
				***BrCHS5*** (Bra020688)
***AtCHI*** (AT3G55120)	***BrCHI1*** (Bra007142)	-	***BrCHI2*** (Bra003209)	***BrCHI3*** (Bra017728)
***AtF3H*** (AT3G51240)	***BrF3H1*** (Bra036828)	***BrF3H2*** (Bra029996)	***BrF3H3*** (Bra012862)	-
***AtF3’H*** (AT5G07990)	***BrF3’H*** (Bra009312)	-	-	-
***AtFLS1*** (AT5G08640)	***BrFLS1*** (Bra009358)	*-*	*-*	-
***AtFSL2*** (AT5G63580)				-
***AtFLS3*** (AT5G63590)	***BrFLS2*** (Bra038647)	***BrFLS3.2*** (Bra029211)	***BrFLS4*** (Bra037747)	
***AtFLS4*** (AT5G63595)	***BrFLS3.1*** (Bra038648)	***BrFLS3.3*** (Bra029212)		
***AtFLS5*** (AT5G63600)				
***AtFLS6*** (AT5G43935)	-	-	*-*	-
*Late biosynthetic genes*				
***AtDFR*** (AT5G42800)	*-*	*-*	***BrDFR*** (Bra027457)	-
***AtANS*** (AT4G22880)	***BrANS1*** (Bra013652)	***BrANS2*** (Bra019350)	*-*	*-*
***AtUGT79B1*** (AT5G54060)	***BrUGT79B1.1*** (Bra003021)	-	*-*	***BrUGT79B1.2*** (Bra035004)
***AtUGT75C1*** (AT4G14090)	-	-	***BrUGT75C1*** (Bra038445)	-
***AtUGT78D2*** (AT5G17050)	-	-	***BrUGT78D2*** (Bra023594)	-
**Regulatory genes (Transcription factor)**				
**Positive regulators**				
*R2R3-MYB*				
*Independent regulatory genes*				
***AtMYB11*** (AT3G62610)	-	**-**	*-*	-
***AtMYB12*** (AT2G47460)	***BrMYB12.1*** (Bra004456)	**-**	***BrMYB12.2*** (Bra000453)	-
***AtMYB111*** (AT5G49330)	***BrMYB111.1*** (Bra037419)	***BrMYB111.2*** (Bra020647)	***BrMYB111.3*** (Bra036145)	-
*Regulation by forming MBW complex*				
***AtPAP1*** (AT1G56650)	-	**-**	**-**	Bra001917^b^
***AtPAP2*** (AT1G66390)	Bra004162^b^	Bra039763^b^	**-**	
***AtMYB113*** (AT1G66370)				
***AtMYB114*** (AT1G66380)				
*bHLH*				
***AtTT8*** (AT4G09820)	***BrTT8*** (Bra037887)	-	**-**	**-**
***AtGL3*** (AT5G41315)	***BrGL3*** (Bra025508)	-	**-**	**-**
***AtEGL3*** (AT1G63650)	*-*	***BrEGL3.1*** (Bra027796)	***BrEGL3.2*** (Bra027653)	**-**
*WD40*				
***AtTTG1*** (AT5G24520)	***BrTTG1.1*** (Bra009770)	***BrTTG1.2*** (Bra029411)	**-**	**-**
**Negative regulators**				
*Single-Repeat R3 MYB*				
***AtMYBL2*** (AT1G71030)	***BrMYBL2.1*** (Bra016164)	***BrMYBL2.2*** (Bra007957)	**-**	**-**
***AtCPC*** (AT2G46410)	***BrCPC1*** (Bra004539)	***BrCPC2*** (Bra039283)	-	**-**
*LATERAL ORGAN BOUNDARY DOMAIN (LBD)*				
***AtLBD37*** (AT5G67420)	***BrLBD37.1*** (Bra012164)	***BrLBD37.2*** (Bra031833)	***BrLBD37.3*** (Bra037847)	-
***AtLBD38*** (AT3G49940)	***BrLBD38.1*** (Bra036040)	*-*	***BrLBD38.2*** (Bra012913)	**-**
***AtLBD39*** (AT4G37540)	***BrLBD39.1*** (Bra011772)	***BrLBD39.2*** (Bra017831)	-	**-**
**Transport genes**				
***AtTT19*** (AT5G17220)	***BrTT19.1*** (Bra008570)	-	***BrTT19.2*** (Bra023602)	**-**

^*a*^Genes in the same grid of the table are in the same tandem array.

^b^High sequence similarity and tandem relationship of these four At R2R3-MYB genes made it difficult to define the three Br genes by synteny or homology analysis. So these three genes were only listed gene code numbers without annotation names.

Among the 73 BrABGs, 67 were syntenic orthologs of 38 AtABGs; only 8.2% of BrABGs had no syntenic relationship. Multiple copies of BrABGs in *B. rapa* syntenic to genes in *A. thaliana* were generated from the WGT, whereas 28 of the 38 AtABGs had less than three syntenic orthologs as a result of gene fractionation following triplication as described above. Furthermore, the amino acid sequence identities for pairs of syntenic genes (80.35%) and non-syntenic genes (80.43%) did not show a statistical difference. The BrABGs of different functions in the anthocyanin biosynthesis pathway had different sequence identities to their counterparts in *A. thaliana* (Additional file 1: Table S1). The homologous pairs of structural genes encoding anthocyanin biosynthesis enzymes shared significantly higher amino acid identity (82.10%) than those encoding transcriptional factors (76.79%), which regulate the expression of structural genes (*P* < 0.05), indicating that the structural genes were more highly conserved than regulatory genes. It is reasonable that excessive mutation of structural genes would likely block the biosynthesis of anthocyanins, reducing the fitness of the plant.

**Figure 1:**
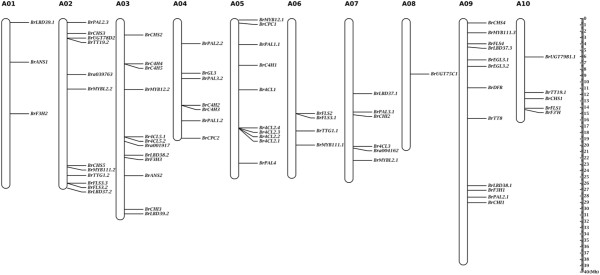
**Distribution of 72 anthocyanin biosynthetic genes (ABGs) on the ten chromosomes of** ***B. rapa.*** The bars indicate the ten chromosomes of *B. rapa* and relative positions of BrABGs were marked on the chromosomes. The scale ruler on the right side showed the physical distance of the chromosomes.

Of the 73 BrABGs, 72 were mapped to the 10 chromosomes of *B. rapa*, with three, 12, 12, seven, 10, four, six, one, 12, and five BrABGs located on chromosomes A01-A10 of *B. rapa* genome V1.5, respectively (Figure 1). The remaining gene, Bra035004, an ortholog of *UGT79B1*, was anchored on Scaffold000100, which has not yet been mapped onto a chromosome. Whole genome analysis established that the three subgenomes in *B. rapa* could be distinguished by the degree of gene density [15, 16]. With this subgenome information, we then assigned all BrABGs to the three subgenomes: LF, MF1 and MF2. In total, 31, 20 and 21 genes were located on LF, MF1 and MF2, respectively. There were more genes located on LF, while almost equal numbers of genes were distributed on MF1 and MF2. Of the 67 syntenic orthologs, 30 were on LF, 17 were on MF1 and 20 were on MF2; the number of syntenic orthologs distributed on LF was a little less than the sum of those on MF1 and MF2. These results show that the distribution of BrABGs is consistent with the gene fractionation status at the whole genome level [15, 16]. Based on the determination of these BrABGs, the anthocyanin biosynthetic pathway in *B. rapa* was thus established.

### ABGs are over-retained in B. rapa

Compared with *A. thaliana*, the *B. rapa* genome has undergone a whole genome triplication [15]. Most of the ABGs were present in multiple copies in *B. rapa*. In our study, the 73 BrABGs represent 0.18% of the 41,174 predicted genes in *B. rapa*; accordingly, there are 41 AtABGs genes representing 0.15% of all *A. thaliana* genes. With the total numbers of *A. thaliana* and *B. rapa* genes as a background, we found that the expansion levels of structural, regulatory, and transport genes and the total number of BrABGs were similar to the whole-genome gene expansion level in *B. rapa* (*P* > 0.05, Table 2). We also analyzed the number and ratio of single copy to multiple copy paralogous genes to reveal the retention status of the BrABGs after the whole genome triplication. We used the ratio of total number of single to multiple copy (two and three) paralogous genes (genes in the same tandem array were only counted once) of the whole *B. rapa* genome as a background (1.77; 15795/8935; [15]) and found that the total BrABGs (0.74; 14/19) and regulatory genes (0.20; 2/10) were significantly lower than that of the background, indicating over-retention of total and regulatory ABGs in *B. rapa*. The structural and transport genes showed no significant difference to the background (Table 3).

**Table 2:** **Comparison of the number of genes involved in the anthocyanin biosynthetic pathways in** ***B. rapa*** **and** ***A. thaliana***

	***A. thaliana*** ^***a***^	***B. rapa***		***P*** value^***b***^
		Syntenic orthologs	Non-syntenic orthologs	
Structural genes	24(1)	41	5	0.3992
Regulatory genes	16(1)	24	1	1
Transport genes	1	2	0	1
Total	41(2)	67	6	0.4414

^*a*^Numbers in brackets refer to genes that have no orthologs in *B. rapa*.

^*b*^The proportion of total *A. thaliana* and *B. rapa* genes was used as a background to calculate the *P*-value using Fisher’s test. A *P*-value more than 0.05 indicates that the proportion of anthocyanin biosynthesis genes between *A. thaliana* and *B. rapa* was not significantly different from the background.

**Table 3:** **Number and ratio of single copy to multiple copy paralogs of AtABGs**

	No. of paralogs with different copies^***a***^					Ratio of single to multiple copies^***b***^	P-value^***c***^
	0	One	Two	Three	Total		
Structural genes	1	12	3	5	21	12:8	0.8987
Regulatory genes	2	2	8	2	14	2:10	0.0019*
Transport genes	0	0	1	0	1	0:1	0.7728
Total	3	14	12	7	36	14:19	0.0173*

^*a*^The number of *A. thaliana* paralogs with different syntenic copies distributed in one to three subgenomes. “0” means the paralogs have no syntenic orthologs in *B. rapa*. Tandemly duplicated genes represent one paralog.

^*b*^The ratio of single to multiple copies is the number of paralogs having one copy versus the total number of paralogs with two and three copies.

^*c*^The proportion of total paralogous sets with different copy numbers over the whole genome was used as a background to calculate the *P*-value using Fisher’s test. The “*” represents a *P*-value less than 0.05, which means the ratio of single to multiple copies of these kinds of anthocyanin pathway genes shows a significant difference from the background.

### The anthocyanin biosynthetic structural genes have expanded through whole genome and tandem duplication in B. rapa

Gene copy numbers can be expanded in four major ways: WGD, tandem duplication (TD), segmental duplication, and gene transposition duplication [19]. We found that the structural BrABGs were expanded through both WGD and TD. We identified 46 BrABG structural genes as homologs of 24 AtABG structural genes. The almost-doubled number of homologs indicated that anthocyanin biosynthetic structural genes were expanded in *B. rapa*. Of the 46 structural genes, 41 were located at 33 loci that had syntenic relationships to 21 AtABG structural gene loci. Among the 41 syntenic orthologs, 14 BrABGs came from six tandem arrays, which accounted for about 30% of all structural genes in *B. rapa*. These data showed that both TD and WGT contributed to the expansion of anthocyanin biosynthetic structural genes in *B. rapa*. The 46 genes were distributed in different subgenomes of *B. rapa*, with 19, 12 and 14 genes located on LF, MF1 and MF2, respectively, and one gene not anchored on any chromosome in the current version of the *B. rapa* genome.

Anthocyanins are derived from branches of the flavonoid pathway, which starts with phenylalanine via the general phenylpropanoid pathway. The phenylpropanoid pathway contains three major genes: *PAL*, *C4H* and *4CL*. Two types of correlated structural genes can be distinguished in the flavonoid biosynthetic pathway: Early Biosynthetic Genes (EBGs) and Late Biosynthetic Genes (LBGs) [20]. The EBGs, which include *CHS*, *CHI*, *F3H*, *F3’H*, and *FLS*, lead to the production of flavonols and other flavonoid compounds, while the LBGs, which include *DFR*, *ANS/LDOX*, and *UFGT*, lead to the production of anthocyanins [9]. The phenylpropanoid pathway genes and EBGs are upstream genes while the LBGs are downstream genes in the anthocyanin biosynthetic pathway. The downstream genes are specifically for anthocyanin biosynthesis [21].

The upstream structural genes have been expanded not only by WGD, but also TD. There are 21 homologs of the nine *A. thaliana* phenylpropanoid pathway genes in *B. rapa* (Table 1). *AtC4H* has five syntenic orthologs in *B. rapa. BrC4H2* and *BrC4H3*, *BrC4H4* and *BrC4H5* are from two tandem arrays located in MF1 and MF2, respectively, while *BrC4H1* is in LF (Table 1). This shows that *BrC4Hs* have expanded by both WGD and TD. *At4CL2* and *At4CL5* have not expanded by whole genome duplication, but their homologs in *B. rapa* form two tandem arrays, with one array containing four genes and the other containing two genes (Table 1).

**Figure 2:**
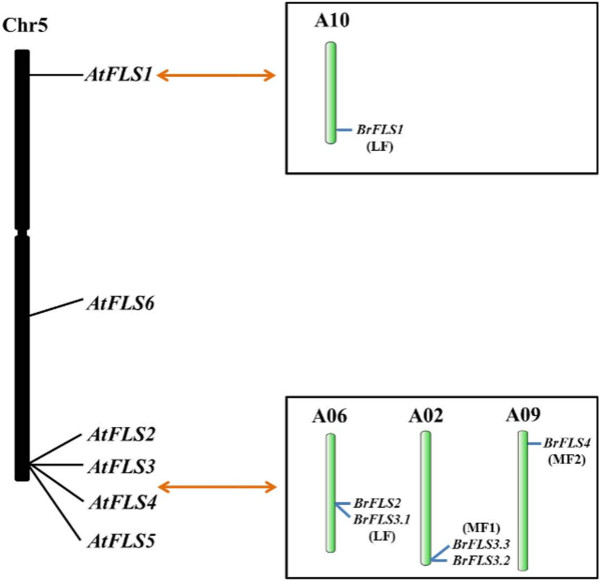
**Arrangement of the** ***AtFLSs*** **in** ***A. thaliana*** **genome as well as their corresponding syntenic homologs in** ***B. rapa*** **chromosomes and subgenomes showed duplication, retention and distribution of** ***BrFLSs.*** All six *AtFLSs* are located on chromosome 5. *AtFLS2* to −*5* are clustered in a 7.5-kb region. *AtFLS1* only has one syntenic ortholog *BrFLS1* on chromosome A10 and subgenome LF. There are no orthologs of *AtFLS6. AtFLS2* to −*5* have five synteny orthologs distributed in different chromosomes and subgenomes. The black and green bars represent the chromosomes of *A. thaliana* and *B. rapa*. The LF, MF1, and MF2 in brackets below gene names indicate the different subgenomes of *B. rapa*.

**Figure 3:**
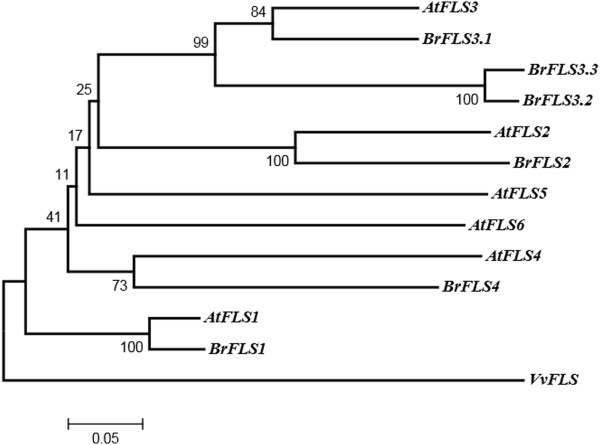
**Phylogeny of the six** ***AtFLS*** **genes, six** ***BrFLS*** **genes, and a** ***FLS*** **gene of** ***Vitis vinifera*** **(wine grape) based on their amino acid sequences.** Bootstrap values (1000 replications) are shown on the branch point. The amino acid sequence of *VvFLS* was used as the outgroup to root the tree.

The biosynthesis of flavonol glycosides and anthocyanins shares common substrates: dihydroflavonols [22]. The synthesis of flavonol aglycones has long been attributed to a single enzyme, flavonol synthase (FLS), which competes with several other enzymes for dihydroflavonol substrates [23]. There are six *FLS* genes in *A. thaliana*: *AtFLS1* to *6*. These six genes are all located on chromosome 5, with *AtFLS2*-*5* arranged in a 7.5-Kb tandem array [21]. There are also six *FLS* genes in *B. rapa*: *BrFLS1*, *BrFLS2*, *BrFLS3.1*, *BrFLS3.2*, *BrFLS3.3*, and *BrFLS4* (Table 1). The tandem array *AtFLS2-5* has syntenic loci in all the three subgenomes of *B. rapa*: *BrFLS2* and *BrFLS3.1* in LF, *BrLFS3.2* and *BrFLS3.3* in MF1, and *BrFLS4* in MF2 (Figure 2). The tandem array was duplicated by WGD, but only some of the four tandemly duplicated genes have been retained, with two, two, and one in LF, MF1 and MF2, respectively (Figure 2). No homologous genes for *AtFLS6* were found in *B. rapa*, neither syntenic nor non-syntenic orthologs. A phylogenetic tree was constructed using the amino acid sequences of the six *AtFLS* genes, the six homologs in *B. rapa* and a *FLS* gene of *Vitis vinifera* to reveal the relationship between these genes (Figure 3). In the phylogenetic tree, *AtFLS1* and *BrFLS1*, *AtFLS2* and *BrFLS2*, *AtFLS3* and *BrFLS3.1*, *3.2*, and *3.3*, and *AtFLS4* and *BrFLS4* were clustered together. These results confirmed the hypothesis proposed by Owens et al. (2008) that the duplications leading to the amplification of the *FLS* gene family were ancient events [21], from at least before the divergence of *B. rapa* and *A. thaliana*.

*DFR*, *LDOX/ANS* and *UFGT*s are the major LBGs (downstream structural genes) of the anthocyanin biosynthetic pathway. DFR (dihydroflavonol-4-reductase) catalyzes the first committed reaction to generate anthocyanins. In *A. thaliana*, the gene *AtDFR* is known to encode a functional DFR [24]. *DFR* was not expanded in *B. rapa*; only one gene, *BrDFR*, was identified as an ortholog of *AtDFR*. Leucoanthocyanidin dioxygenase/anthocyanidin synthase (LDOX/ANS) catalyzes the formation of anthocyanidin, the first colored compound in the anthocyanin biosynthetic pathway [25]. Two syntenic orthologs in *B. rapa*, *BrANS1* and *BrANS2*, were identified by comparative genomic analysis with *A. thaliana*.

The gene copy number ratios between *B. rapa* and *A. thaliana* were 2.3 (21:9), 2 (18:9) and 1.4 (7:5) for the phenylpropanoid pathway genes, EBGs and LBGs, respectively. In addition to WGT, some phenylpropanoid pathway genes and EBGs (which are the upstream structural genes) were also expanded by TD (Table 1). These results show that more upstream structural genes were retained than downstream structural genes of the anthocyanin biosynthetic pathway. The redundancy of the upstream genes may guarantee products for successful downstream anthocyanin synthesis.

### More negative regulatory genes are retained in the anthocyanin biosynthesis regulatory system of B. rapa

Anthocyanin biosynthesis is regulated mostly by the coordinated transcriptional control of the structural genes. The transcriptional control of ABG structural genes has been intensively studied [7]. In *A. thaliana*, anthocyanin biosynthetic regulatory genes can be divided into two groups: positive and negative regulatory genes. For positive regulation of anthocyanin biosynthesis in *A. thaliana*, the spatial and temporal expression of structural genes is mainly determined by R2R3-MYB, basic helix-loop-helix (bHLH) and WD40-type transcriptional factors and their interaction [26]. Four R2R3-MYB (PAP1, PAP2, MYB113 and MYB114) transcription factors and three bHLH (TT8, GL3 and EGL3) proteins combine with the WD40 repeat protein (TTG1) to form ternary transcriptional complexes that activate several anthocyanin biosynthetic structural genes, especially in the later steps (LBGs) of the flavonoid pathway [27–30]. Other R2R3-MYB proteins (MYB11, MYB12, and MYB111) regulate the structural genes of the anthocyanin biosynthetic pathway independently, especially the early steps (EBGs) of the flavonoid pathway [31, 32]. Two single-repeat R3-MYB transcription factors, MYBL2 and CPC (CAPRICE), and three members of the LATERAL ORGAN BOUNDARY DOMAIN (LBD) gene family, LBD37, LBD38, and LBD39, are negative regulators of anthocyanin biosynthesis in *A. thaliana*[33–36]. The regulatory genes in anthocyanin biosynthetic pathway of *B. rapa* were identified as well as the duplication and retention of these genes were analyzed. Detailed information on these biosynthetic regulatory genes in *B. rapa* is presented below*.*

*MYB11*, *MYB12*, and *MYB111* activate EBGs of the anthocyanin biosynthetic pathway in *A. thaliana*[37]. In *B. rapa*, there were two syntenic orthologs of *AtMYB12* and three syntenic orthologs of *AtMYB111* (Table 1), but no homologs of *AtMYB11* were found. These results show that the homolog of *AtMYB11* was lost, while the homologs of *AtMYB12* and *AtMYB111* were over-retained after the WGT of *B. rapa*.

**Figure 4:**
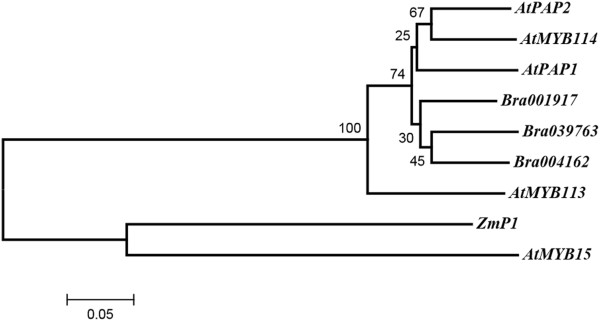
**Phylogenetic tree of** ***AtPAP1,*** ***AtPAP2,*** ***AtMYB113,*** ***AtMYB114,*** ***AtMYB15,*** ***ZmP1,*** **Bra039763, Bra004162 and Bra001917.** Bootstrap values (1000 replications) are shown on the branch point. The amino acid sequence of *ZmP1* was used as the outgroup to root the tree.

*AtPAP1*, *AtPAP2*, *AtMYB113*, and *AtMYB114* (the four R2R3-MYB transcription factors) activate the LBGs by forming ternary complexes with bHLH and WD40 proteins. These four genes are all distributed on chromosome 1 of *A. thaliana. AtPAP2*, *AtMYB113* and *AtMYB114* are present as a three-gene tandem array. The four AtABGs have three orthologs in *B. rapa*: Bra004162, Bra039763 and Bra001917. The high sequence similarity and tandem relationship of these At R2R3-MYB genes made it difficult to name the three Br genes by synteny or homology analysis. Bra004162 and Bra037763 are two syntenic orthologs of the three-gene tandem array. Tandem arrays were not detected in *B. rapa*, indicating that the tandem duplication may have occurred in *A. thaliana* after its divergence from *B. rapa*, or that the redundant tandem arrays were lost through the influence of WGT in *B. rapa*[38]. These data suggest that genes encoding R2R3-MYBs, which form MBW complexes and regulate anthocyanin biosynthesis, were lost after the WGT in *B. rapa* compared with those in *A. thaliana*. Bra001917 is a non-syntenic ortholog of *AtPAP1*, *AtPAP2*, *AtMYB113*, and *AtMYB114*, but its syntenic gene in *A. thaliana* is AT3G23250, which encodes *AtMYB15*. A phylogenetic tree was constructed by aligning the amino acid sequences of *AtPAP1*, *AtPAP2*, *AtMYB113*, *AtMTB114*, Bra001917, Bra004162, Bra039763 and *Zmp1* (a gene encoding a R2R3-MYB transcriptional factor in maize as the outgroup, Figure 4). In the phylogenetic tree, the four *A. thaliana* genes and three *B. rapa* genes are clustered together in one branch, while *AtMYB15* and *Zmp1* form another branch.

Another important group of transcriptional factors is the basic helix-loop-helix (bHLH) gene family, which regulates anthocyanin biosynthesis through formation of MBW ternary complexes. In *A. thaliana*, the bHLH transcriptional factors TT8 (TRANSPARENT TESTA 8), GL3 (GLABROUS 3) and EGL3 (ENHANCER OF GLABRA 3) interact with TTG1 (WD40 protein) and PAP1, PAP2, MYB113, or MYB114 to form multiple MBW complexes that then activate LBGs. In *B. rapa*, there are four *BrbHLH* genes, *BrTT8*, *BrGL3*, *BrEGL3.1* and *BrEGL3.2*, corresponding to three *bHLH* genes in *A. thaliana*. The *BrbHLH* genes in *B. rapa* seem to have undergone relatively extensive fractionation after WGT.

**Figure 5:**
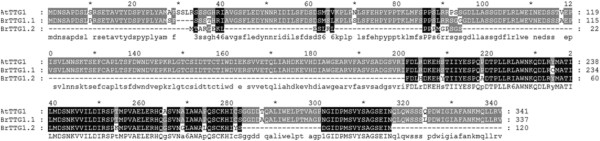
**Amino acid sequences alignment and comparison of the genes** ***AtTTG1,*** ***BrTTG1.1*** **and** ***BrTTG1.2.*** Identical residues are highlighted on a black background, and similar residues are highlighted on a gray background.

*TTG1* (*Transparent Testa Glabra 1*) is the only gene that encodes a WD40 protein involved in the regulation of anthocyanin biosynthesis in *A. thaliana*[39]. In *B. rapa*, there are two paralogous genes, *BrTTG1.1* and *BrTTG1.2*, which are syntenic orthologs of *AtGTT1*. The amino acid sequence of *BrTTG1.2* is shorter than those of *BrTTG1.1* and *AtTTG1. BrTTG1.2* has lost its first WD40 repeat domain, N-terminal and C-terminal regions (Figure 5). A previous study showed that the C-terminal region was vital for the structural and function of TTG1 [40]. A mutant of *A. thaliana* lacking 25 amino acid residues at the C-terminus showed a severe phenotype with no anthocyanins in the testa [40]. These results suggest that *BrTTG1.2* might be non-functional because of large sequence deletions and has become a pseudogene after WGT.

There are several transcription factors including two R3-type single MYB proteins, MYBL2 and CPC, and three N/NO_3_^−^ induced members of the LBD gene family that act as negative regulators of anthocyanin biosynthesis in *A. thaliana. AtMYBL2* is a transcriptional repressor that negatively regulates anthocyanin biosynthesis by interacting with TT8 and MBW complexes [33, 34]. In *B. rapa*, there are two syntenic orthologs, *BrMYBL2.1* and *BrMYBL2.2*, found in LF and MF1, respectively. *AtCPC*, another single repeat R3-MYB transcription factor, works as a negative regulator of anthocyanin biosynthesis [35]. The *AtCPC* gene has two syntenic orthologs in *B. rapa*, *BrCPC1* and *BrCPC2*, which both share more than 90% amino acid sequence identity with their At ortholog. *LBD37*, *LBD38* and *LBD39* negatively regulate the late anthocyanin-specific steps by repressing PAP1 and PAP2 under N/NO_3_^−^ induction [36]. There are seven syntenic orthologs of *AtLBD37*, *AtLBD38* and *AtLBD39* in *B. rapa* (Table 1).

The regulatory genes have expanded mainly through WGT in *B. rapa*. In total, 25 BrABG regulatory genes were identified as homologs of 16 AtABG regulatory genes. Fourteen BrABG positive regulatory genes were identified as homologs of 11 AtABGs, while 11 BrABG negative regulatory genes were identified as homologs of 5 AtABGs. The copy numbers of the positive regulatory genes did not show a significant change between *B. rapa* and *A. thaliana*, but the number of negative regulatory genes was almost doubled in *B. rapa*. Comparing the copy numbers of the regulatory genes, we found that more negative than positive regulatory genes were retained in the *B. rapa* genome.

Anthocyanins play important roles in responding to abiotic and biotic stresses for plants. *B. rapa* comprises a variety of vegetables with rich morphological diversity. Several varieties accumulate variant kinds and contents of anthocyanins in different tissues, while more varieties of *B. rapa* present green with no anthocyanins accumulated (data not shown in this paper). So the copy numbers of negative and positive regulatory genes will help us to understand the metabolic characteristics of anthocyanin in *B. rapa*.

## Conclusions

Anthocyanin biosynthetic genes (ABGs) were identified based on whole-genome comparative analysis between *A. thaliana* and *B. rapa*. Anthocyanin biosynthetic pathways including 73 genes were established in *B. rapa*. Multiple copies of the BrABGs were generated by WGD and retained synteny with their orthologs in *A. thaliana*, but most genes appeared to comprise less than three copies because of gene loss following WGT. More upstream structural genes of the anthocyanin biosynthetic pathway have been retained than downstream. Based on the presence of these homologous structural genes, the anthocyanin biosynthetic pathway in *B. rapa* was then established. More negative regulatory genes have been retained than positive by comparing the copy numbers. The composition of BrABGs could help us to explain the metabolic profiles of anthocyanin accumulation and elaborate the genetic mechanism of anthocyanin biosynthesis in *B. rapa*.

There has been little research on anthocyanin biosynthetic genes in *B. rapa*. Here, we identified anthocyanin biosynthetic genes systematically at the whole genome level. The determination of a complete set of anthocyanin biosynthetic genes in *B. rapa* provides a valuable resource for the study of anthocyanin-related traits and genetic improvement of the anthocyanin nutritional quality of *B. rapa*.

## Methods

### Database for ABGs identification in B. rapa

The complete sets of gene sequences in *A. thaliana* involved in the anthocyanin biosynthetic pathway were downloaded from the TAIR database (http://www.arabidopsis.org/). The *B. rapa* genome sequence (version 1.5) and gene sequences from BRAD (http://brassicadb.org/brad/) [41] were used to identify the ABGs in *B. rapa*.

### Identification of homologous genes between *B. rapa* and *A. thaliana*

We used the anthocyanin biosynthetic gene and protein sequences of *A. thaliana* to align with the genome and protein sequences of *B. rapa* using BLASTN and BLASTP with a cut off E-value ≤ 1E^−10^ and coverage ≥ 0.75. We identified syntenic orthologs between *A. thaliana* and *B. rapa* from BRAD (http://brassicadb.org/brad/), which were determined by both sequence similarity (cutoff: E ≤ 10^−20^ ) and the collinearity of flanking genes [42].

### Phylogenetic analysis of gene sequences

A phylogenetic tree was constructed by the neighbor-joining method using MEGA4 [43]. The stability of tree nodes was tested by bootstrap analysis with 1000 iterations.

### Availability of supporting data

All the anthocyanin biosynthetic genes of *A. thaliana* referred in this paper were retrieved from the TAIR database (http://www.arabidopsis.org/).

The *B. rapa* genome sequence (version 1.5), as well as CDS and protein sequences of BrABGs were retrieved from the *Brassica* database (BRAD) (http://brassicadb.org/brad/).

The protein sequences of *VvFLS* (BAE75808) and *ZmP1* (P27898) used as outgroups to construct the phylogenetic trees in this paper were downloaded from GeneBank (NCBI) (http://www.ncbi.nlm.nih.gov/).

## Electronic supplementary material

Additional file 1: Table S1: Gene inventory of the anthocyanin pathway and the *B. rapa* orthologs. (DOCX 67 KB)

## Abbreviations

LF: Least fractionated
MF1: Medium fractionated
MF2: Most fractionated
WGD: Whole genome duplication
WGT: Whole genome triplication
ABGs: Anthocyanin biosynthetic genes
TD: Tandem duplication
EBGs: Early biosynthetic genes
LBGs: Late biosynthetic genes
bHLH: basic helix-loop-helix
MBW: MYB-bHLH-WD40
PAL: Phenylalanine ammonia-lyase
C4H: Cinnamate-4-hydroxylase
4CL: 4-coumarate:CoA ligase
CHS: Chalcone synthase
CHI: Chalcone isomerase
F3H: Flavanone 3-hydroxylase
F3’H: Flavonoid 3’-hydroxylase
FLS: Flavonol synthase
DFR: Dihydroflavonol-4-reductase
LDOX: Leucoanthocyanidin dioxygenase
ANS: Anthocyanidin synthase
UGT79B1: Anthocyanin 3-O-glucoside: 2 -O-xylosyltransferase
UGT75C1: Anthocyanin 5-O-glucosyltransferase
UGT78D2: Anthocyanidin 3-O-glucosyltransferase
PAP1/2: Production of anthocyanin pigment 1/2
TT8/19: Transparent testa 8/19
GL3: Glabrous 3
EGL3: Enhancer of glabrous 3
TTG1: Transparent testa glabrous 1
MYBL2: MYB-like 2
CPC: CAPRICE
LBD: Lateral organ boundary domain.
