# Supplementary material for: Anthocyanin biosynthetic genes in Brassica rapa
Source: BMC Genomics. 2014 Jun 4;15(1):426. doi: 10.1186/1471-2164-15-426 (PMC4072887; doi:10.1186/1471-2164-15-426)
Supplement: Supplementary file 1 — Additional file 1: Table S1: Gene inventory of the anthocyanin pathway and the B. rapa orthologs. (DOCX 67 KB) [file 12864_2013_6154_MOESM1_ESM.docx]

## Additional file 1: Table S1. Gene inventory of the anthocyanin pathway and the *B. rapa* orthologs.

| **Name** | **AGI No.** | **Gene Annotation** | **Br Name** | **BrID^a^** | **Blocks^b^** | **Subgenome** | | | | | **Identity (Protein)** | | **References** |  |
| --- | --- | --- | --- | --- | --- | --- | --- | --- | --- | --- | --- | --- | --- | --- |
| **Structural genes** | | | | | | | | | | | |  | |  |
| *Biosynthetic genes in phenylpropanoid pathway* | | | | | | | | | | | |  | |  |
| *PAL1* | AT2G37040 | Phenylalanine ammonia-lyase 1 | *BrPAL1.1* | Bra005221 (s) | J (A05) | LF | | | | | 96.35 | | [[1](#_ENREF_1)] |  |
|  |  |  | *BrPAL1.2* | Bra017210 (s) | J (A04) | MF1 | | | | | 94.90 | |  |  |
| *PAL2* | AT3G53260 | Phenylalanine ammonia-lyase 2 | *BrPAL2.1* | Bra006985 (s) | N (A09) | LF | | | | | 91.56 | | [[2](#_ENREF_2)] |  |
|  |  |  | *BrPAL2.2* | Bra039777 (s) | N (A04) | MF1 | | | | | 86.81 | |  |  |
|  |  |  | *BrPAL2.3* | Bra003126 (s) | N (A07) | MF2 | | | | | 59.68 | |  |  |
| *PAL3* | AT5G04230 | Phenylalanine ammonia-lyase 3 | *BrPAL3.1* | Bra028793 (s) | R (A02) | MF2 | | | | | 71.90 | | [[3](#_ENREF_3)] |  |
|  |  |  | *BrPAL3.2* | Bra030322 | I (A04) | MF1 | | | | | 83.64 | |  |  |
| *PAL4* | AT3G10340 | Phenylalanine ammonia-lyase 4 | *BrPAL4* | Bra029831 (s) | F (A05) | LF | | | | | 90.68 | | [[4](#_ENREF_4)] |  |
| *C4H* | AT2G30490 | Cinnamate-4-hydroxylase | *BrC4H1* | Bra018311 (s) | J (A05) | LF | | | | | 95.83 | | [[5](#_ENREF_5)] |  |
|  |  |  | *BrC4H2 BrC4H3* | Bra021636 (s) Bra021637T | J (A04) | MF1 | | | | | 89.26  95.23 | |  |  |
|  |  |  | *BrC4H4 BrC4H5* | Bra022802 (s) Bra022803T | J (A03) | MF2 | | | | | 88.27  95.24 | |  |  |
| *4CL1* | AT1G51680 | 4-coumarate:CoA ligase 1 | *Br4CL1* | Bra030429 (s) | C (A05) | MF2 | | | | | 91.05 | | [[6](#_ENREF_6)] |  |
| *4CL2* | AT3G21240 | 4-coumarate:CoA ligase 2 | *Br4CL2.1*  *Br4CL2.2*  *Br4CL2.3*  *Br4CL2.4* | Bra031262 (s)  Bra031263T  Bra031265T  Bra031266T | F (A05) | LF | | | | | 58.55  63.60  65.81  86.05 | | [[6](#_ENREF_6)] |  |
| *4CL3* | AT1G65060 | 4-coumarate:CoA ligase 3 | *Br4CL3* | Bra004109 (s) | E (A07) | LF | | | | | 88.11 | | [[6](#_ENREF_6)] |  |
| *4CL5* | AT3G21230 | 4-coumarate:CoA ligase 5 | *Br4CL5.1*  *Br4CL5.2* | Bra001819 (s) Bra001820T | F (A03) | MF2 | | | | | 86.62  80.03 | | [[6](#_ENREF_6)] |  |
| *CHS* | AT5G13930 | Chalcone synthase | *BrCHS1* | Bra008792 (s) | R (A10) | LF | | | | | 95.44 | | [[7](#_ENREF_7)] |  |
|  |  |  | *BrCHS2* | Bra006224 (s) | R (A03) | MF1 | | | | | 94.44 | |  |  |
|  |  |  | *BrCHS3* | Bra023441 (s) | R (A02) | MF2 | | | | | 94.18 | |  |  |
|  |  |  | *BrCHS4* | Bra036307 | O (A09) | LF | | | | | 89.57 | |  |  |
|  |  |  | *BrCHS5* | Bra020688 | Wa (A02) | MF1 | | | | | 88.10 | |  |  |
| *CHI* | AT3G55120 | Chalcone isomerase | *BrCHI1* | Bra007142 (s) | N (A09) | LF | | | | | 81.50 | | [[7](#_ENREF_7)] |  |
|  |  |  | *BrCHI2* | Bra003209 (s) | N (A07) | MF2 | | | | | 71.95 | |  |  |
|  |  |  | *BrCHI3* | Bra017728 | U (A03) | MF1 | | | | | 73.16 | |  |  |
| *F3H* | AT3G51240 | Flavanone 3-hydroxylase | *BrF3H1* | Bra036828 (s) | N (A09) | LF | | | | | 93.02 | | [[8](#_ENREF_8)] |  |
|  |  |  | *BrF3H2* | Bra029996 (s) | N (A01) | MF1 | | | | | 34.46 | |  |  |
|  |  |  | *BrF3H3* | Bra012862 (s) | N (A03) | MF2 | | | | | 91.21 | |  |  |
| *F3'H* | AT5G07990 | Flavonoid 3'-hydroxylase | *BrF3'H* | Bra009312 (s) | R (A10) | LF | | | | | 85.99 | | [[9](#_ENREF_9)] |  |
| *FLS1* | AT5G08640 | Flavonol synthase 1 | *BrFLS1* | Bra009358 (s) | R (A10) | LF | | | | | 91.96 | | [[10](#_ENREF_10)] |  |
| *FSL2*  *FLS3*  *FLS4*  *FLS5* | AT5G63580  AT5G63590  AT5G63595  AT5G63600 | Flavonol synthase 2  Flavonol synthase 3  Flavonol synthase 4  Flavonol synthase 5 | *BrFLS2*  *BrFLS3.1*  *BrFLS3.2*  *BrFLS3.3*  *BrFLS4* | Bra038647 (s)  Bra038648T  Bra029211 (s)  Bra029212T  Bra037747 (s) | X (A06)  X (A02)  X (A09) | LF  MF1  MF2 | | | | | 74.90  61.88  66.01  67.76  58.61 | | [[11](#_ENREF_11)] |  |
| *FLS6* | AT5G43935 | Flavonol synthase 6 | *BrFLS6* | - | - | - | | | | | - | | [[11](#_ENREF_11)] |  |
| *DFR* | AT5G42800 | Dihydroflavonol-4-reductase | *BrDFR* | Bra027457 (s) | V (A09) | | MF2 | | | | 86.23 | | [[12](#_ENREF_12)] |  |
| *ANS (LDOX)* | AT4G22880 | Leucoanthocyanidin dioxygenase | *BrANS1* | Bra013652 (s) | U (A01) | | LF | | | | 91.29 | | [[10](#_ENREF_10)] |  |
|  |  |  | *BrANS2* | Bra019350 (s) | U (A03) | | MF1 | | | | 91.60 | |  |  |
| *UGT79B1* | AT5G54060 | Anthocyanin 3-O-glucoside: 2-O-xylosyltransferase | *BrUGT79B1.1* | Bra003021 (s) | Wb (A10) | | LF | | | | 87.50 | | [[13](#_ENREF_13)] |  |
|  |  |  | *BrUGT79B1.2* | Bra035004 | - | | - | | | | 86.93 | |  |  |
| *UGT75C1* | AT4G14090 | Anthocyanin 5-O-glucosyltransferase | *BrUGT75C1* | Bra038445 (s) | T (A08) | | MF2 | | | | 79.30 | | [[14](#_ENREF_14)] |  |
| *UGT78D2* | AT5G17050 | Anthocyanidin 3-O-glucosyltransferase | *BrUGT78D2* | Bra023594 (s) | R (A02) | | MF2 | | | | 79.87 | | [[15](#_ENREF_15)] |  |
| **Regulatory genes (Transcription factor)** | | | | | | | | | | | |  | |  |
| **Positive regulators** | | | | | | | | | | | |  | |  |
| ***R2R3-MYB*** | | | | | | | | | | | |  | |  |
| *Independent regulatory genes* | | | | | | | | | | | |  | |  |
| *MYB11* | AT3G62610 | MYB Domain Protein 11 |  | - | - | | | - | |  | | | [[16](#_ENREF_16), [17](#_ENREF_17)] |  |
| *MYB12* | AT2G47460 | MYB Domain Protein12 | *BrMYB12.1* | Bra004456 (s) | J (A05) | | | LF | | 68.45 | | | [[16](#_ENREF_16), [17](#_ENREF_17)] |  |
|  |  |  | *BrMYB12.2* | Bra000453 (s) | J (A03) | | | MF2 | | 74.02 | | |  |  |
| *MYB111* | AT5G49330 | MYB Domain Protein111 | *BrMYB111.1* | Bra037419 (s) | Wa (A06) | | | LF | | 77.62 | | | [[16](#_ENREF_16), [17](#_ENREF_17)] |  |
|  |  |  | *BrMYB111.2* | Bra020647 (s) | Wa (A02) | | | MF1 | | 61.07 | | |  |  |
|  |  |  | *BrMYB111.3* | Bra036145 (s) | Wa (A09) | | | MF2 | | 73.78 | | |  |  |
| *PAP1(MYB75)* | AT1G56650 | Production of Anthocyanin Pigment 1 |  | Bra039763 | E (A02) | | MF1 | | | 77.51 | | | [[18](#_ENREF_18)] |  |
|  |  |  |  | Bra001917 | F (A03) | | MF2 | | | 75.90 | | |  |  |
|  |  |  |  | Bra004162 | E (A07) | | LF | | | 73.06 | | |  |  |
| *PAP2(MYB90)* | AT1G66390 | Production of Anthocyanin Pigment 2 |  | Bra004162 (s) | E (A07) | | LF | | | 74.39 | | | [[18](#_ENREF_18)] |  |
|  |  |  |  | Bra001917 | F (A03) | | MF2 | | | 76.00 | | |  |  |
|  |  |  |  | Bra039763 (s) | E (A02) | | MF1 | | | 76.00 | | |  |  |
| *MYB113* | AT1G66370 | MYB Domain Protein 113 |  | Bra039763 (s) | E (A02) | | MF1 | | | 69.72 | | | [[18](#_ENREF_18)] |  |
|  |  |  |  | Bra004162 (s) | E (A07) | | LF | | | 66.80 | | |  |  |
| *MYB114* | AT1G66380 | MYB Domain Protein 114 |  | Bra039763 (s) | E (A02) | | MF1 | | | 85.51 | | | [[18](#_ENREF_18)] |  |
|  |  |  |  | Bra004162 (s) | E (A07) | | LF | | | 86.33 | | |  |  |
| ***bHLH*** | | | | | | | | | | | |  | |  |
| *TT8* | AT4G09820 | Transparent Testa 8 | *BrTT8* | Bra037887 (s) | P (A09) | | LF | | | 73.08 | | | [[19](#_ENREF_19), [20](#_ENREF_20)] |  |
| *GL3* | AT5G41315 | Glabrous 3 | *BrGL3* | Bra025508 (s) | S (A04) | | LF | | | 84.64 | | | [[21](#_ENREF_21)] |  |
| *EGL3* | AT1G63650 | Enhancer of Glabrous 3 | *BrEGL3.1* | Bra027796 (s) | D (A09) | | MF1 | | | 85.83 | | | [[21](#_ENREF_21)] |  |
|  |  |  | *BrEGL3.2* | Bra027653 (s) | D (A09) | | MF2 | | | 83.97 | | |  |  |
| ***WD40*** | | | | | | | | | | | |  | |  |
| *TTG1* | AT5G24520 | Transparent Testa Glabrous 1 | *BrTTG1.1* | Bra009770 (s) | Q (A06) | LF | | | 92.96 | | | | [[22](#_ENREF_22)] |  |
|  |  |  | *BrTTG1.2* | Bra029411 (s) | Q (A02) | MF1 | | | 75.00 | | | |  |  |
| **Negative regulators** | | | | | | | | | | | |  | |  |
| ***Single-Repeat R3 MYB*** | | | | | | | | | | | |  | |  |
| *MYBL2* | AT1G71030 | MYB-Like 2 | *BrMYBL2.1* | Bra016164 (s) | E (A07) | LF | | | 71.07 | | | | [[23](#_ENREF_23)] |  |
|  |  |  | *BrMYBL2.2* | Bra007957 (s) | E (A02) | MF1 | | | 71.13 | | | |  |  |
| *CPC* | AT2G46410 | CAPRICE | *BrCPC1* | Bra004539 (s) | J (A05) | LF | | | 92.94 | | | | [[24](#_ENREF_24)] |  |
|  |  |  | *BrCPC2* | Bra039283 (s) | J (A04) | MF1 | | | 90.59 | | | |  |  |
| ***LATERAL ORGAN BOUNDARY DOMAIN (LBD)*** | | | | | | | | | | | |  | |  |
| *LBD37* | AT5G67420 | LOB Domain-containing Protein 37 | *BrLBD37.1* | Bra012164 (s) | X (A07) | LF | | | 83.72 | | | | [[25](#_ENREF_25)] |  |
|  |  |  | *BrLBD37.2* | Bra031833 (s) | X (A02) | MF1 | | | 76.77 | | | |  |  |
|  |  |  | *BrLBD37.3* | Bra037847 (s) | X (A09) | MF2 | | | 74.90 | | | |  |  |
| *LBD38* | AT3G49940 | LOB Domain-containing Protein 38 | *BrLBD38.1* | Bra036040 (s) | M (A09) | LF | | | 74.80 | | | | [[25](#_ENREF_25)] |  |
|  |  |  | *BrLBD38.2* | Bra012913 (s) | M (A03) | MF2 | | | 73.41 | | | |  |  |
| *LBD39* | AT4G37540 | LOB Domain-containing Protein 39 | *BrLBD39.1* | Bra011772 (s) | U (A01) | LF | | | 76.42 | | | | [[25](#_ENREF_25)] |  |
|  |  |  | *BrLBD39.2* | Bra017831 (s) | U (A03) | MF1 | | | 71.95 | | | |  |  |
| **Transport genes** | | | | | | | | | | | |  | |  |
| *TT19* | AT5G17220 | Transparent Testa 19 | *BrTT19.1* | Bra008570 (s) | R (A10) | LF | | | 84.51 | | | | [[26](#_ENREF_26), [27](#_ENREF_27)] |  |
|  |  |  | *BrTT19.2* | Bra023602 (s) | R (A02) | MF2 | | | 84.98 | | | |  |  |

^a^ “(s)”indicates BrABGs that exhibit synteny. “T” indicates genes in the same tandem array as the above genes.

^b^ Letters refer to 24 conserved blocks (A–X) that represent conserved segments identifiable in the ancestral karyotype, *A. thaliana* and *B. rapa* [[28](#_ENREF_28)]. Two letters indicate that the gene is located at the boundary between two blocks; text in parentheses indicates the specific chromosome of *B. rapa*. Bra035004 was anchored on Scaffold000100, which has not yet been mapped onto a chromosome.

# References

1. Ohl S, Hedrick SA, Chory J, Lamb CJ: **Functional properties of a phenylalanine ammonia-lyase promoter from Arabidopsis**. *The Plant Cell Online* 1990, **2**(9):837-848.

2. Shufflebottom D, Edwards K, Schuch W, Bevan M: **Transcription of two members of a gene family encoding phenylalanine ammonia‐lyase leads to remarkably different cell specificities and induction patterns**. *The Plant Journal* 1993, **3**(6):835-845.

3. Wanner LA, Li G, Ware D, Somssich IE, Davis KR: **The phenylalanine ammonia-lyase gene family in Arabidopsis thaliana**. *Plant molecular biology* 1995, **27**(2):327-338.

4. Cochrane FC, Davin LB, Lewis NG: **The< i> Arabidopsis</i> phenylalanine ammonia lyase gene family: kinetic characterization of the four PAL isoforms**. *Phytochemistry* 2004, **65**(11):1557-1564.

5. Mizutani M, Ohta D, Sato R: **Isolation of a cDNA and a genomic clone encoding cinnamate 4-hydroxylase from Arabidopsis and its expression manner in planta**. *Plant Physiology* 1997, **113**(3):755-763.

6. Ehlting J, Büttner D, Wang Q, Douglas CJ, Somssich IE, Kombrink E: **Three 4‐coumarate: coenzyme A ligases in Arabidopsis thaliana represent two evolutionarily divergent classes in angiosperms**. *The Plant Journal* 1999, **19**(1):9-20.

7. Shirley BW, Kubasek WL, Storz G, Bruggemann E, Koornneef M, Ausubel FM, Goodman HM: **Analysis of Arabidopsis mutants deficient in flavonoid biosynthesis**. *The Plant Journal* 1995, **8**(5):659-671.

8. Pelletier MK, Shirley BW: **Analysis of flavanone 3-hydroxylase in Arabidopsis seedlings (Coordinate regulation with chalcone synthase and chalcone isomerase)**. *Plant physiology* 1996, **111**(1):339-345.

9. Schoenbohm C, Martens S, Eder C, Forkmann G, Weisshaar B: **Identification of the Arabidopsis thaliana flavonoid 3'-hydroxylase gene and functional expression of the encoded P450 enzyme**. *Biological chemistry* 2000, **381**(8):749-753.

10. Pelletier M, Murrell J, Shirley B: **Characterization of flavonol synthase and leucoanthocyanidin dioxygenase genes in Arabidopsis. Further evidence for differential regulation of "early" and "late" genes**. *Plant physiology* 1997, **113**(4):1437-1445.

11. Owens DK, Alerding AB, Crosby KC, Bandara AB, Westwood JH, Winkel BS: **Functional analysis of a predicted flavonol synthase gene family in Arabidopsis**. *Plant physiology* 2008, **147**(3):1046-1061.

12. Shirley BW, Hanley S, Goodman HM: **Effects of ionizing radiation on a plant genome: analysis of two Arabidopsis transparent testa mutations**. *The Plant Cell Online* 1992, **4**(3):333-347.

13. Yonekura‐Sakakibara K, Fukushima A, Nakabayashi R, Hanada K, Matsuda F, Sugawara S, Inoue E, Kuromori T, Ito T, Shinozaki K: **Two glycosyltransferases involved in anthocyanin modification delineated by transcriptome independent component analysis in Arabidopsis thaliana**. *The Plant Journal* 2012, **69**(1):154-167.

14. Tohge T, Nishiyama Y, Hirai M, Yano M, Nakajima J-i, Awazuhara M, Inoue E, Takahashi H, Goodenowe D, Kitayama M *et al*: **Functional genomics by integrated analysis of metabolome and transcriptome of Arabidopsis plants over-expressing an MYB transcription factor**. *The Plant journal : for cell and molecular biology* 2005, **42**(2):218-235.

15. Yin R, Messner B, Faus-Kessler T, Hoffmann T, Schwab W, Hajirezaei M-R, von Saint Paul V, Heller W, Schäffner AR: **Feedback inhibition of the general phenylpropanoid and flavonol biosynthetic pathways upon a compromised flavonol-3-O-glycosylation**. *Journal of experimental botany* 2012, **63**(7):2465-2478.

16. Stracke R, Ishihara H, Huep G, Barsch A, Mehrtens F, Niehaus K, Weisshaar B: **Differential regulation of closely related R2R3‐MYB transcription factors controls flavonol accumulation in different parts of the Arabidopsis thaliana seedling**. *The Plant Journal* 2007, **50**(4):660-677.

17. Stracke R, Jahns O, Keck M, Tohge T, Niehaus K, Fernie AR, Weisshaar B: **Analysis of PRODUCTION OF FLAVONOL GLYCOSIDES‐dependent flavonol glycoside accumulation in Arabidopsis thaliana plants reveals MYB11‐, MYB12‐and MYB111‐independent flavonol glycoside accumulation**. *New Phytologist* 2010, **188**(4):985-1000.

18. Stracke R, Werber M, Weisshaar B: **The< i> R2R3-MYB</i> gene family in< i> Arabidopsis thaliana</i>**. *Current opinion in plant biology* 2001, **4**(5):447-456.

19. Zhang F, Gonzalez A, Zhao M, Payne CT, Lloyd A: **A network of redundant bHLH proteins functions in all TTG1-dependent pathways of Arabidopsis**. *Development* 2003, **130**(20):4859-4869.

20. Nesi N, Debeaujon I, Jond C, Pelletier G, Caboche M, Lepiniec L: **The TT8 gene encodes a basic helix-loop-helix domain protein required for expression of DFR and BAN genes in Arabidopsis siliques**. *The Plant Cell Online* 2000, **12**(10):1863-1878.

21. Bernhardt C, Lee MM, Gonzalez A, Zhang F, Lloyd A, Schiefelbein J: **The bHLH genes GLABRA3 (GL3) andENHANCER OF GLABRA3 (EGL3) specify epidermal cell fate in the Arabidopsis root**. *Development* 2003, **130**(26):6431-6439.

22. Gonzalez A, Zhao M, Leavitt JM, Lloyd AM: **Regulation of the anthocyanin biosynthetic pathway by the TTG1/bHLH/Myb transcriptional complex in Arabidopsis seedlings**. *The Plant Journal* 2008, **53**(5):814-827.

23. Dubos C, Le Gourrierec J, Baudry A, Huep G, Lanet E, Debeaujon I, Routaboul JM, Alboresi A, Weisshaar B, Lepiniec L: **MYBL2 is a new regulator of flavonoid biosynthesis in Arabidopsis thaliana**. *The Plant Journal* 2008, **55**(6):940-953.

24. Zhu H-F, Fitzsimmons K, Khandelwal A, Kranz RG: **CPC, a single-repeat R3 MYB, is a negative regulator of anthocyanin biosynthesis in Arabidopsis**. *Molecular Plant* 2009, **2**(4):790-802.

25. Rubin G, Tohge T, Matsuda F, Saito K, Scheible W-R: **Members of the LBD family of transcription factors repress anthocyanin synthesis and affect additional nitrogen responses in Arabidopsis**. *The Plant Cell Online* 2009, **21**(11):3567-3584.

26. Kitamura S, Shikazono N, Tanaka A: **TRANSPARENT TESTA 19 is involved in the accumulation of both anthocyanins and proanthocyanidins in Arabidopsis**. *The Plant journal : for cell and molecular biology* 2004, **37**(1):104-114.

27. Sun Y, Li H, Huang J-R: **Arabidopsis TT19 functions as a carrier to transport anthocyanin from the cytosol to tonoplasts**. *Molecular plant* 2012, **5**(2):387-400.

28. Schranz ME, Lysak MA, Mitchell-Olds T: **The ABC's of comparative genomics in the Brassicaceae: building blocks of crucifer genomes**. *Trends in plant science* 2006, **11**(11):535-542.
